# Galectin-9 promotes a suppressive microenvironment in human cancer by enhancing STING degradation

**DOI:** 10.1038/s41389-020-00248-0

**Published:** 2020-07-06

**Authors:** Chuan-xia Zhang, Dai-jia Huang, Valentin Baloche, Lin Zhang, Jing-xiao Xu, Bo-wen Li, Xin-rui Zhao, Jia He, Hai-qiang Mai, Qiu-yan Chen, Xiao-shi Zhang, Pierre Busson, Jun Cui, Jiang Li

**Affiliations:** 1 Department of Biotherapy, State Key Laboratory of Oncology in South China, Collaborative Innovation Center for Cancer Medicine, Guangdong Key Laboratory of Nasopharyngeal Carcinoma Diagnosis and Therapy, Sun Yat-sen University Cancer Center, and School of Life Sciences, Sun Yat-sen University, 510060 Guangzhou, P. R. China; 2 MOE Key Laboratory of Gene Function and Regulation, State Key Laboratory of Biocontrol, Sun Yat-sen University, 510275 Guangzhou, China; 3 Department of Nasopharyngeal Carcinoma, Sun Yat-sen University Cancer Center, 510060 Guangzhou, China; 4 CNRS, UMR 9018, Gustave Roussy and Université Paris-Saclay 39 rue Camille Desmoulins, F-94805 Villejuif, France; 5 Department of Neurosurgery, The First People’s Hospital of Changzhou, Changzhou, 213000 Jiangsu China; 6 Department of Research and Development, Shenzhen Institute for Innovation and Translational Medicine, Shenzhen International Biological Valley-Life Science Industrial Park, Dapeng New District, Shenzhen, China

**Keywords:** Cancer microenvironment, Tumour immunology

## Abstract

Galectin-9 (Gal-9) is known to enhance the expansion of myeloid-derived suppressor cells (MDSCs) in murine models. Its contribution to the expansion of MDSCs in human malignancies remain to be investigated. We here report that Gal-9 expression in nasopharyngeal carcinoma (NPC) cells enhances the generation of MDSCs (CD33^+^CD11b^+^HLA-DR^−^) from CD33^+^ bystander cells. The underlying mechanisms involve both the intracellular and secreted Gal-9. Inside carcinoma cells, Gal-9 up-regulates the expression of a variety of pro-inflammatory cytokines which are critical for MDSC differentiation, including IL-1β and IL-6. This effect is mediated by accelerated STING protein degradation resulting from direct interaction of the Gal-9 carbohydrate recognition domain 1 with the STING C-terminus and subsequent enhancement of the E3 ubiquitin ligase TRIM29-mediated K48-linked ubiquitination of STING. Moreover, we showed that extracellular Gal-9 secreted by carcinoma cells can enter the myeloid cells and trigger the same signaling cascade. Consistently, high concentrations of tumor and plasma Gal-9 are associated with shortened survival of NPC patients. Our findings unearth that Gal-9 induces myeloid lineage-mediated immunosuppression in tumor microenvironments by suppressing STING signaling.

## Introduction

Galectins are an intriguing family of β-galactoside-binding animal lectins, which have multiple functions inside the cells but can also be secreted by unconventional pathways independently of the classical endoplasmic reticulum/Golgi trafficking machinery^1^. Extra-cellular galectin-9 (Gal-9) was originally described as an eosinophil chemoattractant^2^. More recently it has been identified as a versatile immune-modulator acting on a wide range of target cells. It is known to induce the apoptosis of effector T-cells through binding with T-cell immunoglobulin and mucin domain-containing molecule 3 (Tim-3)^3^. In contrast, extra-cellular Gal-9 enhances the differentiation and suppressive activity of regulatory T cells, and is involved in dendritic cell (DC) maturation^1^. Intra-cellular Gal-9 also has multiple functions. It is involved in intracellular trafficking, cell adhesion and migration, proliferation and apoptosis^4–6^.

In the past 5 years, various immunosuppressive effects of Gal-9 have been reported for several types of human malignancies, for example its induction of M2 polarization of macrophages in melanoma^7,8^. However, the contribution of Gal-9 to the generation and expansion of MDSCs in human malignancies is barely known. Dardalhon et al. have reported an excess of CD11b^+^Ly6G^+^ MDSCs in transgenic mice overexpressing Gal-9 or Tim-3. A role for extra-cellular Gal-9 binding the Tim-3 receptor was supported by the abrogation of Gal-9 effect on MDSC expansion in Tim-3 knockout (KO) mice. However, this report provides no clue on whether or not intra-cellular Gal-9 is also involved in the promotion of MDSCs^9^. Moreover, to our knowledge, no similar investigations have been made on human MDSCs, especially in the context of malignant tumors. To address these questions, we returned to the model of nasopharyngeal carcinoma (NPC) for two main reasons. Firstly, our previous studies showed that the expansion of the MDSC population in peripheral blood and tumor tissues from NPC patients was associated with a more aggressive tumor behavior^10,11^. Secondly, we and others have shown that Gal-9 concentrations are at a high level in malignant NPC cells in situ, especially in recurrent tumors, in NPC-derived PDX (patient-derived xenograft model) as well as in plasma exosomes from NPC patients^12–14^, suggesting a role of Gal-9 in tumor progression. In addition, NPC is a major public health problem in various countries especially Southern China. While malignant NPC cells consistently express Epstein–Barr virus latent antigens, the NPC microenvironment is heavily infiltrated by various kinds of inflammatory cells^15^. Until now, the cellular and viral mechanisms of tumor immune escape have not been fully understood.

Myeloid-derived suppressor cells (MDSCs), neutrophils, and DCs have been implicated in T-cell suppression in a wide range of malignancies. They are recruited in the tumor microenvironment and educated by tumor cells to create a localized immunosuppressive niche for tumor survival^16,17^. Generally, the tumor-promoting myeloid response is linked to the environmental cytokine and chemokine release. For instance, the high levels of IL-1β, IL-6, and CXCL2 in the tumor microenvironment favor MDSC expansion^18,19^. Recent studies showed that innate immune regulators including stimulator of interferon genes (STING) and toll-like receptors (TLR) are involved in the regulation of myeloid cell generation and differentiation in cancers^20,21^. However, the molecular mechanisms that contribute to the rise of tumor-promoting myeloid responses in the cancer microenvironment is complex and still enigmatic.

To explore the biological functions of intra- and extra-cellular Gal-9 in regulating myeloid cell differentiation in human cancer, we used in vitro experimental systems based on the co-cultivation of CD33^+^ peripheral leucocytes with NPC cells engineered to express and release various amounts of Gal-9. Using these models, we demonstrated that both intra-cellular and extra-cellular Gal-9 are key players for the promotion of immunosuppressive myeloid cells in the tumor microenvironment. To a large extent, this effect is supported by down-regulation of the STING protein inside Gal-9-producing carcinoma cells. Furthermore, we have confirmed that high amounts of Gal-9 in the tumor tissue and the serum from tumor-bearing patients is associated with a pejorative outcome.

## Results

### Forced Gal-9 expression in malignant NPC cells promotes a cytokine profile conducive to myeloid cell differentiation

**Fig. 1:**
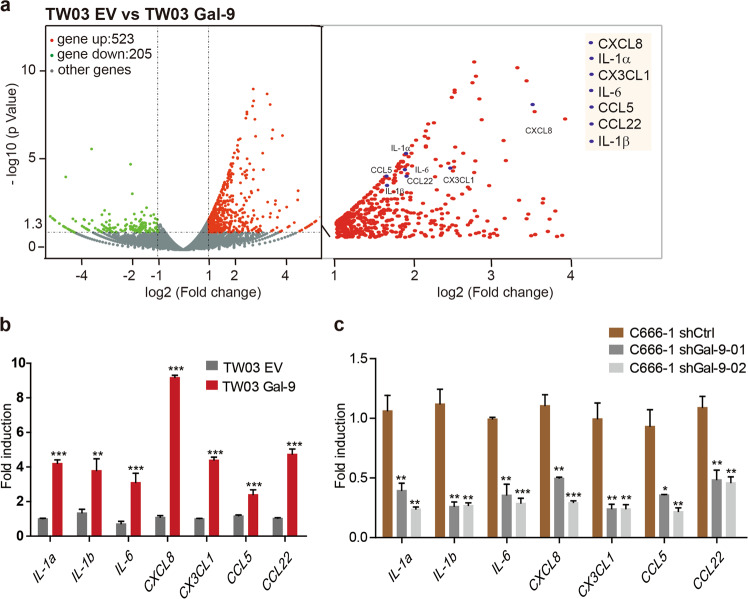
Gal-9 expression in NPC cells modulates the expression of genes related to innate immune cell differentiation. **a** RNA sequencing was performed on stably transfected TW03 cells with or without Gal-9 overexpression, TW03-Gal-9 (Gal-9 vector) and TW03-EV (empty vector) respectively. The differentially expressed genes were selected according to the criteria of *p* value < 0.05 and fold change >2. Differentially expressed genes are visualized on a Volcano plot. The mRNA levels of indicated genes were determined, using quantitative real time PCR (qRT-PCR), in four distinct cell types: TW03-Gal-9 and TW03-EV cells (**b**) and Gal-9 knockdown (shGal9-01 and shGal9-02) or control C666-1 cells (shCtrl) (**c**). The experiments in (**b**, **c**) were performed at least three times, and the data were plotted as the mean ± SEM. Statistics were conducted with an unpaired Student’s *t* test, *p < 0.05 vs. the corresponding control. NS not significant, EV Empty vector.

To understand the molecular characteristics of malignant cell with endogenous overexpression of Gal-9, we set up an experimental system based on transfected NPC cells allowing in vitro modulation of Gal-9 expression. Firstly, we assessed the baseline protein expression of Gal-9 in different NPC cell lines TW03 (EBV negative, low Gal-9 expression) and C666-1 (EBV-positive, high Gal-9 expression) (Supplementary Fig. S1a). From parental NPC cells, we generated Gal-9 stably overexpressed or knockdown NPC cells including TW03-Gal-9 and C666-1-sh-Gal-9 and their corresponding control cells including TW03-EV (empty vector) and C666-1-sh-control cells for further studies (Supplementary Fig. S1b, c). We employed a full transcriptome analysis to identify differentially regulated genes and pathways in the TW03-Gal-9 and TW03-EV cells. A total of 728 genes were differentially regulated between control (EV) and Gal-9 overexpression cells (523 genes up-regulated vs. 205 genes down-regulated). Pathway analysis revealed that many of these altered genes are involved in cytokine-cytokine receptor interactions (Supplementary Fig. S1d). Further analyses of the gene transcription profiles showed that a series of genes encoding cytokines and chemokines such as CXCL8, IL-1α, CX3CL1, IL-6, CCL5, CCL22, and IL-1β, were significantly up-regulated (*p* < 0.0001; Fig. 1a). We further verified that the mRNA levels of *CXCL8*, *IL-1α*, *CX3CL1*, *IL-6*, *CCL5*, *CCL22*, and *IL-1β* were increased in TW03-Gal-9 cells, while decreased in C666-1-shGal-9-01 and C666-1-shGal-9-02 cells compared with the corresponding control cells (Fig. 1b, c). Overall, these data suggested that Gal-9 mainly up-regulated the production of a subset of cytokines involved in myeloid cell differentiation, especially IL-1β and IL-6.

### Intracellular and extracellular Gal-9 expressed by malignant cells promotes tumor-associated MDSC differentiation in a cytokine-induced manner

**Fig. 2:**
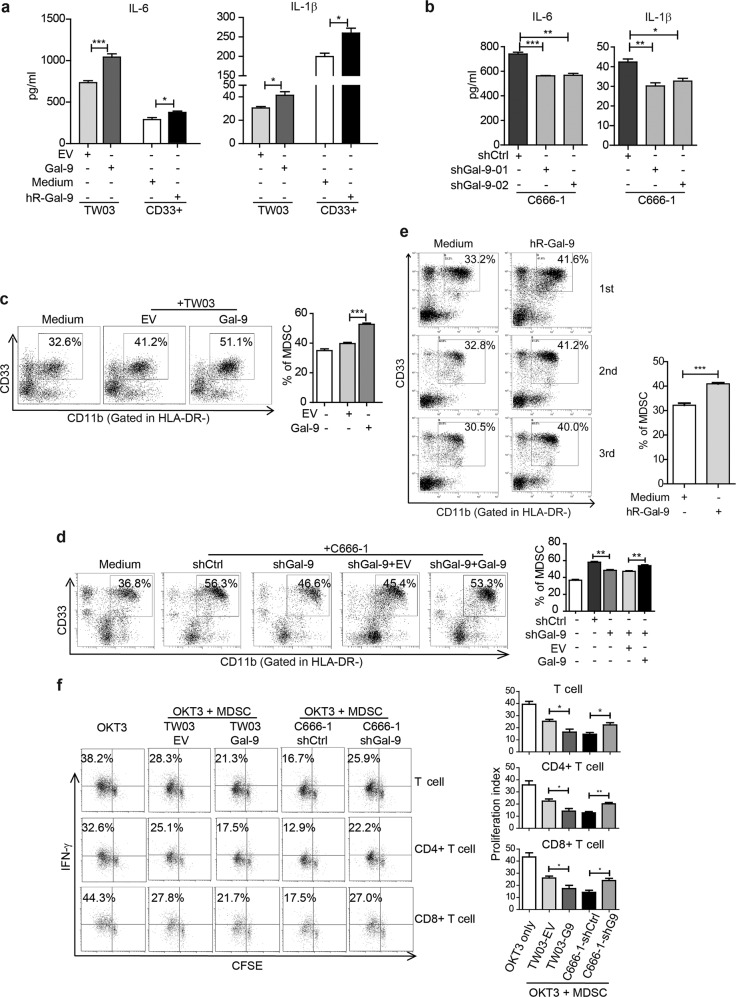
The effect of intra- and extra-cellular Gal-9 on MDSC differentiation and expansion in vitro. ELISA assay of IL-6 and IL-1β concentrations in the conditioned media of TW03-EV and TW03-Gal-9 cells or CD33^+^ cells (**a**) or Gal-9 knockdown (shGal9-01 and shGal9-02) and control C666-1 cells (**b**) with or without addition of human recombinant Gal-9 (hR-Gal-9). **c** Differentiation of CD33^+^ cells isolated from healthy PBMCs investigated after treatment with control medium, co-cultivation with TW03-EV or TW03-Gal-9. Representative flow cytometry plots (**left**) and histogram (**right**) of MDSC differentiation assays showing the percentage of CD33^+^CD11b^+^ cells in the HLA-DR^−^ gate arising from CD33^+^ cells. **d** Similar experiment based on co-cultivation with C666-1 cells using four experimental conditions: C666-1-shCtrl cells (shCtrl), unmodified C666-1-shGal9-01 (shGal-9), C666-1-shGal9-01 co-transfected with a control lentiviral vector (shGal9 + EV) or a Gal-9 lentiviral vector (ShGal-9+Gal-9). **e** Representative flow cytometry plots (**left**) and histogram (**right**) of MDSC differentiation assays showing the percentage of CD33^+^CD11b^+^ cells in the HLA-DR^−^ gate arising from CD33^+^ cells with or without addition of human recombinant Gal-9 (hR-Gal-9). **f** Representative flow cytometry plots (**left**) and histogram (**right**) of the IFN-γ-positive, proliferating T cells (CSFE assay) to test the immune-suppressive activity of MDSC cells induced by TW03-EV and TW03-Gal-9 or C666-1-shCtrl and C666-1-shGal9 (shGal9-01) cells. All experiments were performed at least three times, and the quantification data were plotted as the mean ± SEM. Statistics were conducted with an unpaired Student’s *t* test, *p < 0.05, **p < 0.01, ***p < 0.001 vs. the corresponding control. NS not significant, EV Empty vector.

Considering that Gal-9 is a secreting protein, here we further assess the repercussions of Gal-9 expression in malignant cells on the regulation of bystander CD33^+^ cells belonging to the human myeloid lineage and the effect of extracellular Gal-9 on CD33^+^ cell function and differentiation using RhGal-9 protein or tumor-derived exosomes (T-EXO) with substantial Gal-9 abundance. Firstly, we found that the production of IL-1β and IL-6 were increased in TW03 and CD33^+^ cells with forced up-regulation of Gal-9 compared with that of corresponding control cells, while decreased in C666-1-shGal-9-01 and C666-1-shGal-9-02 cells compared with the corresponding control cells (Fig. 2a, b). In a previous work, we have shown that IL-1β and IL-6 promote myeloid cell generation and differentiation in vitro^10^. Therefore, for in vitro mimicking of the tumor microenvironment, we used a Transwell co-culture system in vitro, connecting carcinoma cells and CD33^+^ myeloid cells. We found that forced Gal-9 expression in TW03 cells significantly enhanced the induction of CD33^+^CD11b^+^HLA-DR^−^ MDSCs from CD33^+^ myeloid lineage cells in the neighborhood of TW03 cells (Fig. 2c). Reciprocally, depletion of Gal-9 in C666-1 cells lowered the induction of CD33^+^CD11b^+^HLA-DR^−^ MDSCs derived from CD33^+^ cells, however forced Gal-9 expression in C666-1-shGal-9 cells was able to restore the same capacity of MDSCs induction as the parental cells (Fig. 2d). Consistently, the treatment of CD33^+^ cells with RhGal-9 resulted in their enhanced differentiation in CD33^+^CD11b^+^HLA-DR^−^MDSC (Fig. 2e). Interestingly, the same effect was achieved when treating CD33^+^ cells with exosomes derived from NPC cells having high Gal-9 production (Supplementary Fig. S2). Simultaneously, the proliferation of IFNγ−producing T cells, including CD4^+^ and CD8^+^ T cells, was strongly suppressed by TW03-Gal-9- induced MDSCs, while the C666-1-shGal-9-induced MDSCs displayed weaker suppression on the proliferation of IFNγ−producing CD4^+^ and CD8^+^ T cells (Fig. 2f). Our data suggest that up-regulation of Gal-9 expression in NPC cells promotes MDSC expansion depending on IL-1β and IL-6 induction from tumor cell and myeloid cell itself.

### Endogenous Gal-9 downregulates STING expression leading to tumor-derived MDSC expansion in a cytokine-dependent manner

**Fig. 3:**
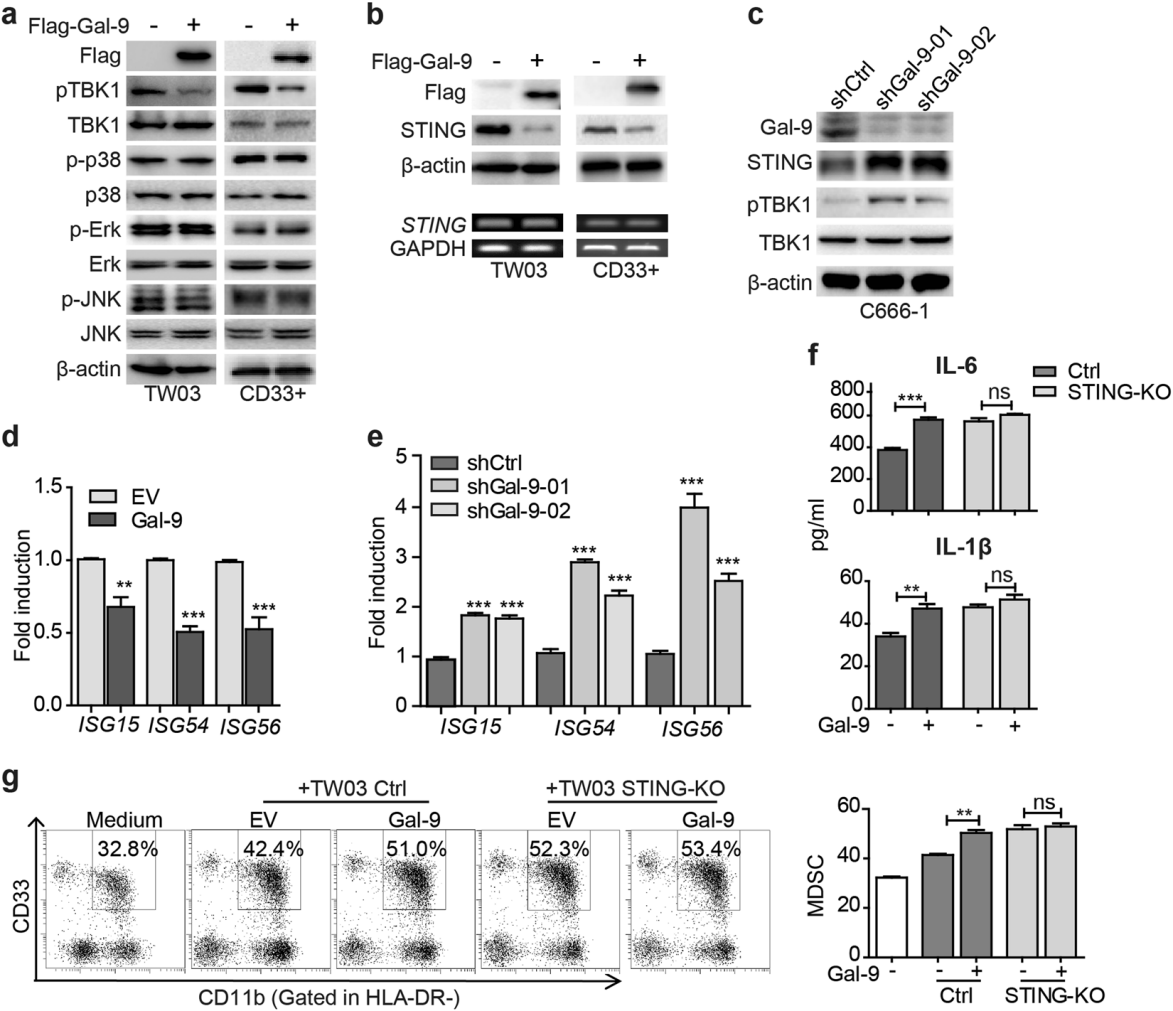
Endogenous Gal-9 downregulates STING leading to tumor-associated MDSC expansion depending on cytokine-induction. **a** The extracts of TW03 cells stably expressing Flag-tagged-EV or Gal-9 (left) and CD33^+^ cells transfected with lenti-flag-tagged-EV or lenti-flagged-Gal-9 vector (right) were subjected to immunoblot with the indicated antibodies. **b** Upper panel: the lysates of TW03 cells and CD33^+^ cells expressing Flag-tagged EV or Gal-9 were subjected to immunoblot with the indicated antibodies. Lower panel: RT-PCR analysis of *STING* mRNA; *GAPDH* mRNA served as a transcript of reference. **c** The lysates of C666-1 cells transfected with Gal-9-specific shRNAs were subjected to immunoblot with the indicated antibodies. The mRNA expression levels of ISG genes were determined in TW03 cells transfected with the Gal-9-expressing vector or EV (**d**) C666-1 cells transfected with shRNA targeting Gal-9 (**e**) or corresponding control vectors using quantitative real time PCR (qRT-PCR). **f** ELISA assay of IL-6 and IL-1β concentrations in the supernatants from control or STING-KO TW03 cells, transfected with Flag-EV and Flag-Gal-9 for 48 h. **g** Representative flow cytometry plots (**left**) and histogram (**right**) of MDSC differentiation assay for CD33^+^ cells co-cultured with Control (Ctrl) or STING-KO TW03 cells, transfected with Flag- EV or Flag-Gal-9 for 48 h. All experiments were performed at least three times, and the quantification data were plotted as the mean ± SEM. Statistics were conducted with an unpaired Student’s *t* test, *p < 0.05, **p < 0.01, ***p < 0.001 vs. the corresponding control. NS not significant, EV empty vector, Ctrl control, KO knock out.

Our next goal was to elucidate how endogenous Gal-9 was able to induce a change in the cytokine profile in NPC cell lines TW03 and C666-1, or CD33^+^ myeloid cells. We investigated the status of several proteins involved in cytokine regulation, including the type I IFN (p-TBK1 and TBK1) and MAPK (p38, JNK, and ERK) signaling pathways. We found that overexpression of Gal-9 specifically inhibited the type I interferon (IFN) signaling pathway, but not the MAPK signaling pathways (Fig. 3a). We further found that STING, the key adapter protein in type I IFN signaling, was down-regulated at the protein level in TW03 and CD33^+^ cells undergoing forced expression of Gal-9 (Fig. 3b). Reciprocally, the STING signaling was up-regulated in C666-1 cells following Gal-9 silencing (Fig. 3c). In addition, the mRNA levels of downstream genes of the STING signaling pathway including *ISG15*, *ISG54*, and *ISG56* were down-regulated in TW03-Gal-9 cells (Fig. 3d), but up-regulated in C666-1-shGal-9-01 and C666-1-shGal-9-02 cells (Fig. 3e). Previously, we have shown that inactivation of STING signaling in NPC cells leads to the tumor-derived MDSC expansion in a cytokine-dependent manner via the STING/SOCS1/STAT3 axis^22^. Thus, these results led us to hypothesize a connection between the biological function of Gal-9 and the STING signaling pathway. We then employed STING knockout NPC cells in additional coculture experiments. Interestingly, we found that the forced expression of Gal-9 in TW03-vector control cells resulted in a consistent increase in their secretion of IL-1β and IL-6, however the Gal-9-mediated increase of IL-1β and IL-6 secretion was disrupted in TW03-STING-KO cells. (Fig. 3f and Supplementary Fig. S3). Consistently, an effect of Gal-9 to promote the generation of MDSCs in the vicinity of TW03 cells was observed in cells retaining STING expression but not in STING-KO cells whose capacity to enhance MDSC differentiation was already close to its maximum (Fig. 3g). These data suggested that Gal-9 promotes tumor-associated MDSC differentiation in a STING-dependent manner.

### Gal-9 interacts with STING

**Fig. 4:**
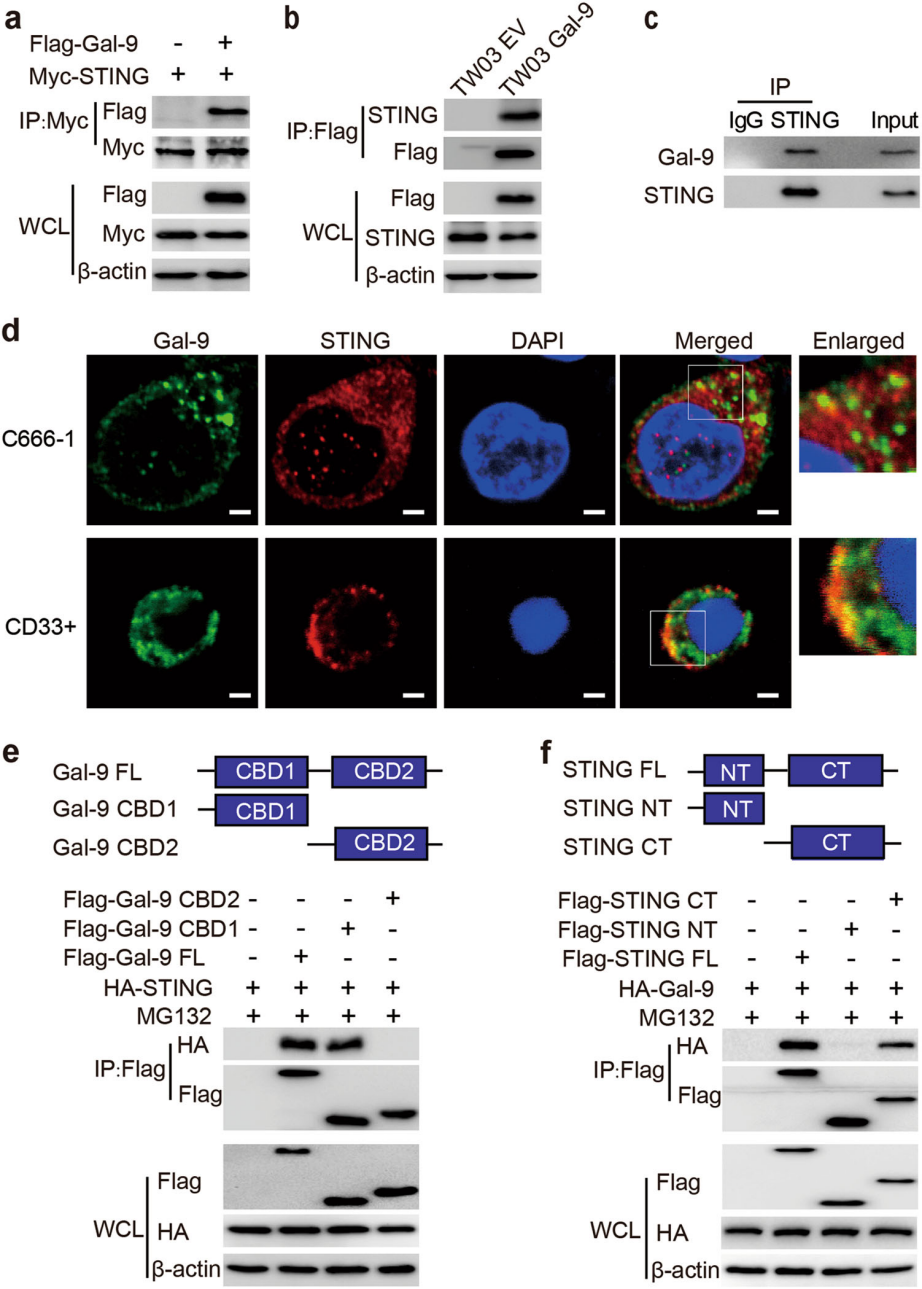
Gal-9 interacts with STING. **a** HEK293T cells were transfected with Flag-Gal-9 or Flag-EV vector together with a Myc-STING expression vector for 24 h. The cell extracts were subjected to immunoprecipitation with anti-Myc-beads and immunoblot analysis with anti-Flag antibody. **b** The lysates of TW03 cells stably expressing Flag- EV or Flag-Gal-9 were subjected to immunoprecipitation with anti-Flag-beads and immunoblot analysis with anti-STING antibody. **c** Extracts of C666-1 cells were subjected to immunoprecipitation with an anti-STING antibody and immunoblot analysis with the anti-Gal-9 antibody. **d** Immunofluorescence assay to detect the colocalization of Gal-9 (green) and STING (red) in C666-1 and CD33^+^ cells. Cell nuclei were stained with DAPI (blue). Scale bar: 2 μm. **e** Top: Schematic diagram of Gal-9 and its domain mutants. Bottom: HEK293T cells transfected with Flag-EV, Flag-Gal-9-FL, Flag-Gal-9-CBD1 and Flag-Gal-9-CBD2 mutants, together with HA-STING for 24 h, then treated with MG132 (10 μM) for 6 h. The extracts from HEK293T cells subjected to immunoprecipitation with anti-Flag-beads and immunoblot analysis with anti-HA antibody. **f** Top: Schematic diagram of STING and its domain mutants. Bottom: HEK293T cells transfected with Flag-EV, Flag-STING-FL, Flag-STING-NT and Flag-STING-CT, together with HA-Gal-9 for 24 h, then treated with MG132 (10 μM) for 6 h. The extracts from HEK293T cells were subjected to immunoprecipitation with anti-Flag-beads and immunoblot analysis with anti-HA antibody. All experiments were performed at least three times. EV empty vector, FL full length, CBD carbohydrate-binding domain, NT N terminal, CT C terminal, WCL whole cell lysis.

Based on the above data (Fig. 3b, c), we wondered whether Gal-9 could suppress the STING protein through a network of protein interactions. We determined that Gal-9 interacted with STING by co-immunoprecipitation of Gal-9 with STING using anti-Myc on HEK293T cell extracts (Fig. 4a). Reciprocally the endogenous STING was co-immunoprecipiatated with Flag-Gal-9 from TW03 extract (Fig. 4b). Finally the endogenous Gal-9 was co-immunoprecipitated with the endogenous STING from wild type C666-1 extracts (Fig. 4c). In addition, we observed that Gal-9 colocalized with STING in the cytoplasm of C666-1 and CD33^+^ cells using immunofluorescent staining (Fig. 4d).

To explore the domain of the Gal-9 protein interacting with STING, we generated two domain-deletion mutants of Gal-9 (Gal-9-CBD1 and Gal-9-CBD2, containing only one carbohydrate-binding domain, respectively). Co-immunoprecipitation analyses showed that STING strongly interacted with Gal-9-CBD1, but not Gal-9-CBD2 (Fig. 4e). Importantly, the CBD1 domain of Gal-9 was also responsible for STING degradation (Supplementary Fig. S4). We next generated two domain-deletion mutants of STING and found that STING interacted with Gal-9 only at its C-terminal domain (Fig. 4f). These data suggested that Gal-9 promoted STING degradation via direct protein-protein interactions.

### Gal-9 enhances the TRIM29-mediated K48-linked ubiquitination of STING by protein-to-protein binding

**Fig. 5:**
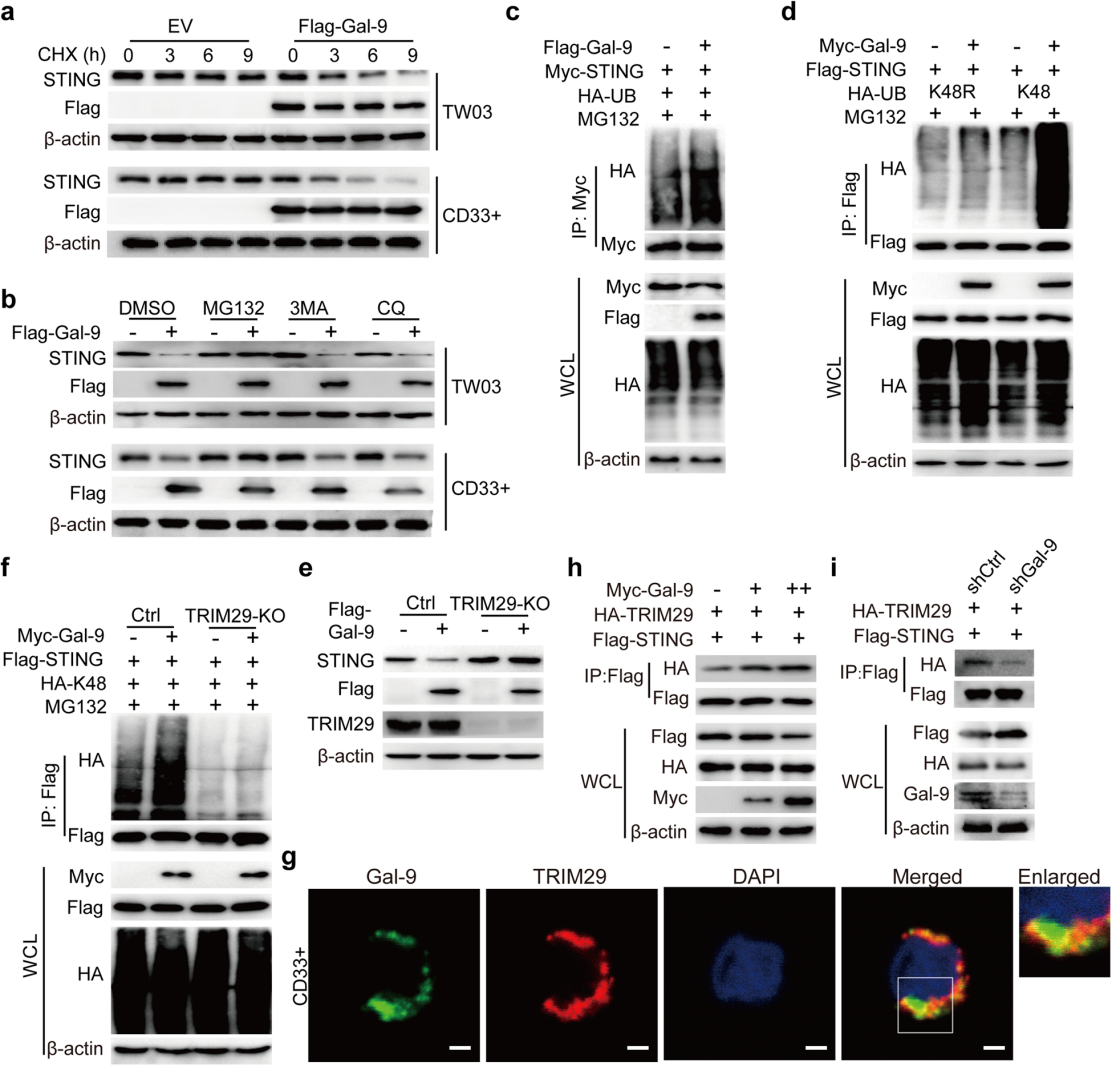
Gal-9 enhances STING interaction with and ubiquitination by TRIM29. **a** Upper panel: TW03 cells stably expressing Flag-Gal-9 or EV were treated with cycloheximide (CHX, 20 μg/ml) for indicated time points (in hours) and then harvested for immunoblot. Lower panel: CD33^+^ cells transfected with lenti-flag-tagged-EV or lenti-flagged-Gal-9 vector for 36 h, then treated with CHX (20 μg/ml) for indicated time points (in hours) and harvested for immunoblot. **b** Upper panel: Immunoblot analysis of protein extracts of TW03 cells stably expressing Flag-Gal-9 or EV, and treated with DMSO, MG132 (10 μM), 3-Methyladenine (3-MA, 2.5 mM) or Chloroquine phosphate (CQ, 20 mM) for 6 h. Lower panel: CD33^+^ cells transfected with lenti-flag-tagged-EV or lenti-flagged-Gal-9 vector for 36 h, then treated with DMSO, MG132 (10 μM), 3-Methyladenine (3-MA, 2.5 mM) or Chloroquine phosphate (CQ, 20 mM) for 6 h and harvested for immunoblot. **c** Protein extracts of HEK293T cells transfected with a combination of plasmids coding for Myc-STING and HA-UB, in the presence or absence of Flag-Gal-9 and subjected to immunoprecipitation with anti-Myc-beads and immunoblot analysis with anti-HA antibody. **d** Protein extracts of HEK293T cells transfected with various combinations of plasmids coding for Flag-STING, Myc-Gal-9, HA-UB-K48, or HA-UB-K48R (arginine instead of lysine 48) and subjected to immunoprecipitation with anti-Flag-beads and immunoblot analysis with indicated antibodies. **e** Control (Ctrl) and TRIM29-KO TW03 cells were transfected with Flag-Gal-9 or EV for 24 h. The cell protein extracts were immunoblotted with the indicated antibodies. **f** Control (Ctrl) and TRIM29-KO TW03 cells were transfected with a combination of plasmids coding for Flag-STING, HA-K48, Myc-Gal-9 for 36 h, and treated with MG132 (10 μM) for 6 h. The extracts from TW03 cells were subjected to IP with an anti-Flag-beads and immunoblot analysis with anti-HA antibody. **g** Immunofluorescence assay demonstrating the partial colocalization of endogenous Gal-9 (green) and TRIM29 (red) in CD33^+^ cells. Cell nuclei were stained with DAPI (blue). Scar bar: 2 μm. **h** HEK293T cells were transfected with Flag-STING and HA-TRIM29 followed by transfection with increasing amounts of Myc-Gal-9 for 24 h. The protein extracts were subjected to IP with anti-Flag-beads and immunoblot analysis with an anti-HA antibody. **i** C666-1 cells were transfected with shRNA targeting Gal-9 (C666-1-shGal-9) or corresponding control vectors (C666-1-shCtrl) for 24 h, then transfected with HA-TRIM29 and Flag-STING for 24 h, finally subjected to IP with anti-Flag-beads and immunoblot with indicated antibodies. All experiments were performed at least three times. Ctrl Control, KO knock out, WCL whole cell lysis, IP Immunoprecipitation.

We further explored the molecular mechanisms of Gal-9-dependent the decrease of STING expression, and found that the half-life of STING was shorter in TW03-Gal-9 cells or lenti-Gal-9-treated CD33^+^ cells than in TW03-EV cells or lenti-Control-treated CD33^+^ cells using cycloheximide (CHX) ‘chase’ assays (Fig. 5a), suggesting that Gal-9 enhance STING protein degradation. Importantly, it is the proteasomal inhibitor MG132 but not 3MA or CQ (lysosomal or autophagic inhibitors) that inhibited the STING protein degradation in TW03-Gal-9 cells or lenti-Gal-9-treated CD33^+^ cells (Fig. 5b). Our observations suggested that the degradation of the STING protein mediated by Gal-9 is dependent on the ubiquitin-proteasome pathway.

We further found that Gal-9 promotes the ubiquitination of STING in TW03 cells (Fig. 5c). It has been reported that K48-linked ubiquitination is typically associated with protein degradation via ubiquitin-proteasome pathway. Here, we demonstrated that Gal-9 specifically promoted K48-linked poly-ubiquitination of STING, but not other ubiquitination linkages (Fig. 5d). Our data indicated that Gal-9 mediates the proteasomal degradation of STING protein by enhancing its K48-linked ubiquitination.

Since Gal-9 is not an E3 ubiquitin ligase, we hypothesize that Gal-9 might recruit certain E3 ligase to mediate the ubiquitination of STING. The E3 ubiquitin ligase tripartite motif containing 29 (TRIM29) has been reported to mediate the K48-linked ubiquitination of STING^23^. Hence, we generated TW03-TRIM29-KO cells (Supplementary Fig. S5a), and found that Gal-9 failed to degrade STING in TW03-TRIM29-KO cells anymore (Fig. 5e). In addition, forced expression of both Gal-9 and TRIM29 resulted in more rapid STING degradation (Supplementary Fig. S5b). Of note, TRIM29 deficiency completely impaired the ability of Gal-9 to induce K48-linked ubiquitination of STING (Fig. 5f). Furthermore, we found that Gal-9 interacted with TRIM29 in CD33^+^ and TW03 cells (Fig. 5g and Supplementary Fig. S5c), and we found that Gal-9 enhanced the interaction between TRIM29 and STING (Fig. 5h, i). Altogether, these observations suggested that Gal-9 recruits TRIM29 to mediate the K48-linked ubiquitination and degradation of STING.

### High abundance of Gal-9 in tumor tissue and serum is associated with a more aggressive phenotype of NPC tumors

**Fig. 6:**
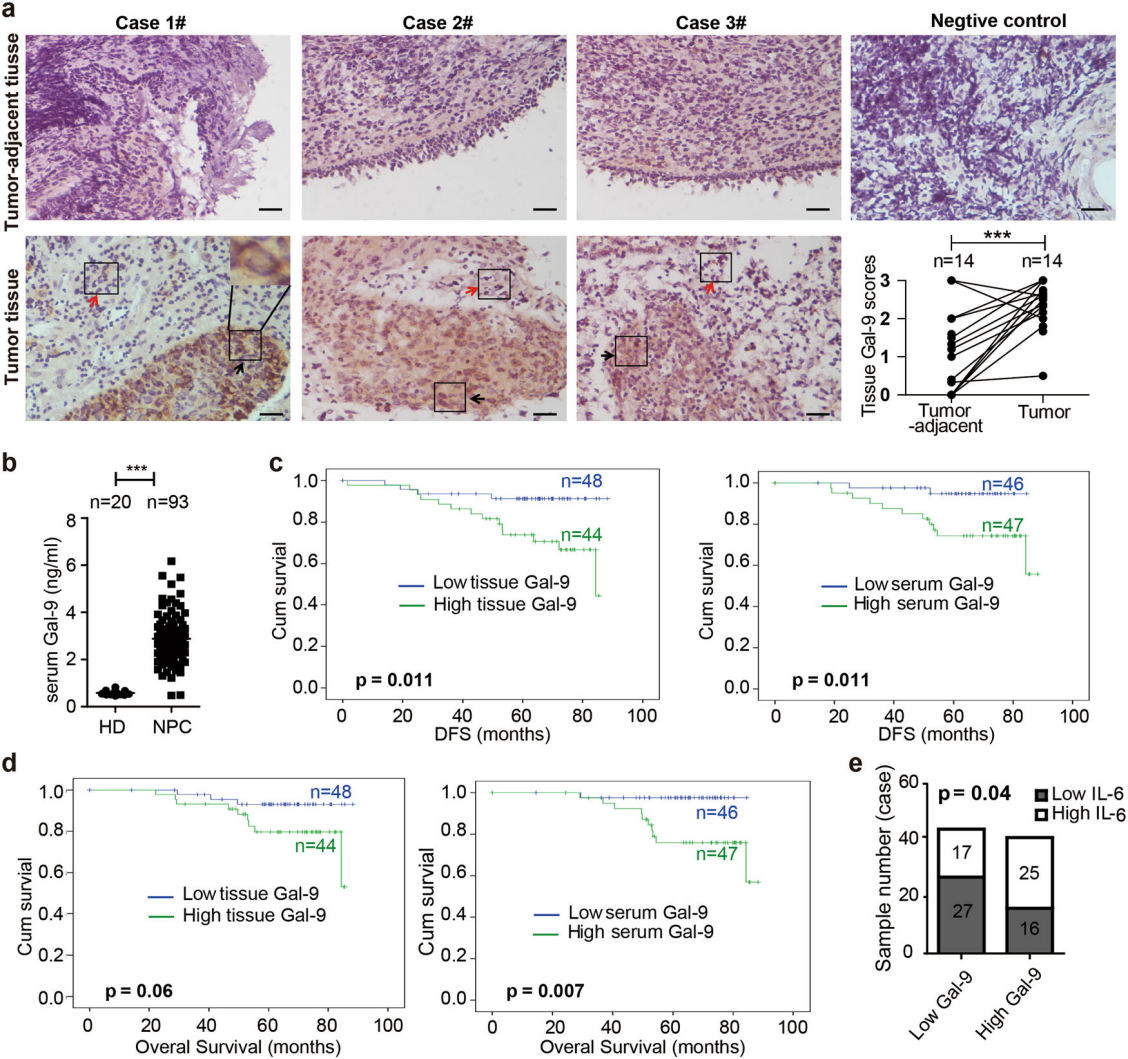
Overexpression of Gal-9 in NPC is associated with an unfavorable outcome. **a** Images of the three left most columns: comparative IHC assessment of in situ Gal-9 expression on tissue sections of NPC tumors (lower panels) and matched tumor-adjacent tissues from the same patients (upper panels). Black arrows and red arrows point to the Gal-9-positive tumor cells and tumor-infiltrated cells, respectively. Upper panel of the right most column: one example of a tumor section stained with an irrelevant primary antibody of the same isotype as the anti-Gal-9. Lower panel of the right most column: results of the semi-quantitative scoring of Gal-9 staining on pairs of tumor/tumor-adjacent tissues from 14 NPC patients, Scale bar: 50 μm. **b** Serum concentrations of Gal-9 in NPC patients and healthy donors assessed by ELISA (*n* = 93 and 20, respectively). **c** Disease-free survival (DFS) rates showing that the intracellular tumor (*n* = 92, *p* = 0.011) and the extracellular serum Gal-9 (*n* = 93, *p* = 0.011) levels were significantly associated with a pejorative outcome of NPC patients (for both tumor and serum Gal-9, the threshold between patients with low and high abundance were the median values of the IHC score and serum concentration respectively). **d** Overall survival (OS) rates showing that the intracellular tumor (*n* = 92, *p* = 0.06) and the extracellular serum Gal-9 (*n* = 93, *p* = 0.007) levels were associated with a poor outcome of NPC patients. **e** Existence of a positive correlation between the serum concentrations of Gal-9 and IL-6 in NPC patients (*n* = 85, *p* = 0.04). Statistical significance of (**a**, **b**) was determined by an unpaired Student’s *t* test, (**c**, **d**) was determined by Kaplan–Meier and log-rank test and (**e**) was determined by a *χ*^2^ test, *p < 0.05, **p < 0.01, ***p < 0.001 and *p* < 0.05 was considered significant.

Whether Gal-9 expressed by malignant cells enhances or attenuates the malignant phenotype remains a subject of controversy^12,24^. To address this issue in the case of NPCs, we performed concomitant assessment of Gal-9 abundance in the primary tumors by IHC on tissue sections and in the peripheral blood by ELISA on plasma samples. Gal-9 staining on tumor sections was followed by semi-quantitative scoring for 92 patients (IHC score). We found that for almost all patients for whom adjacent nonmalignant tissue was available, Gal-9 was consistently more abundant in tumor tissues including tumor cells and tumor-infiltrated cells than in surrounding non-tumor tissues (*p* < 0.001, *n* = 14, Fig. 6a). By ELISA, we also made a comparative assessment of the Gal-9 concentration in 93 serum samples from NPC patients and samples from 20 healthy donors. Gal-9 serum concentrations were much greater for NPC patients (*p* < 0.001, Fig. 6b). The abundance of Gal-9 in the tumor and, even more, in the serum was associated with a less favorable outcome (shorter DFS and OS for patients with high IHC score in the tumor sections and high concentrations of Gal-9 in the serum, Fig. 6c, d). As shown in Table S1, there were no significant correlations between the IHC score and clinical parameters. On the other hand, serum Gal-9 concentrations were correlated with the clinical extension stage and consistently with the stage of lymph nodes (Table S1). Importantly, consistent with the aforementioned in vitro observations, we found that IL-6 concentrations were positively correlated to Gal-9 concentrations in serum samples from NPC patients (*p* < 0.05, Fig. 6e). Overall, these observations were compatible with the idea that the production of intracellular and extracellular Gal-9 by carcinoma cells promotes tumor development.

## Discussion

Immunotherapies targeting immune checkpoint molecules, including PD1, CTLA-4, and Tim-3 have been a major breakthrough in cancer therapy and have changed our understanding of host-tumor interactions^25,26^. However, in most types of human malignancies, only a fraction of the patients, generally about 20–30%, achieved a good response to the current immune checkpoint inhibitors^27,28^. Therefore, there is a need to characterize factors of immune suppression distinct from the classical checkpoints PD1 and CTLA4. There is strong evidence that extra-cellular Gal-9 is one of them. Indeed, extra-cellular Gal-9 has an almost constant immunosuppressive function in vitro and in murine models. It has been shown that extracellular Gal-9 contributes to tumor growth in several syngeneic murine tumor models^29^. In several human malignancies, high concentrations of Gal-9 in plasma are associated with a more aggressive tumor phenotype^30,31^. However, a distinct picture emerges when dealing with Gal-9 detected in tumor tissue sections. There is evidence that high abundance of Gal-9 in tumor sections is associated with a better prognosis for a number of human malignancies for example gastric and mammary carcinomas^8,32^. To address this paradox, it is important to keep in mind that most Gal-9 detected in tissue sections is probably intra-cellular and that intra-cellular Gal-9 is expected to decrease cell motility and tumor invasion^32^. However, in the case of NPC, high abundance of Gal-9 in tissue sections has been reported to be associated with more aggressive diseases^12^. This is compatible with the hypothesis that in some cellular backgrounds, even intra-cellular Gal-9 can have oncogenic activity or favor an immunosuppressive activity^30^. To address this last hypothesis, we have compared the cytokine profiles of NPC cells with various abundances of intra-cellular Gal-9. This set of experiments has shown that intra-cellular Gal-9 as well as extra-cellular Gal-9 induces production of cytokines, which enhance MDSC expansion. For memory, MDSCs have multiple deleterious effects in tumor progression. In addition to their immunosuppressive effects, they can contribute to angiogenesis and even have a direct impact on the proliferation of malignant cells through their secretion of growth factors^33^.

**Fig. 7:**
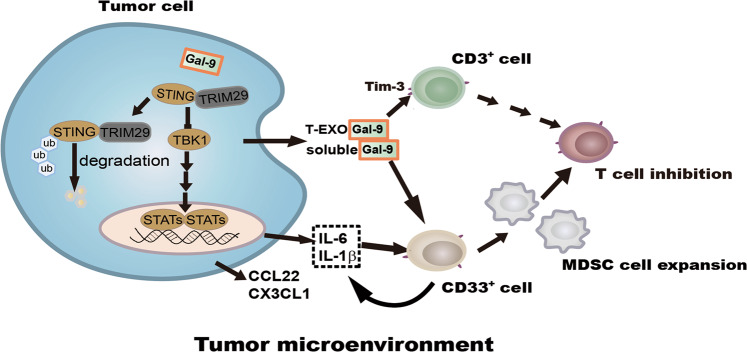
A cartoon recapitulating the contribution of intracellular and extracellular tumor Gal-9 to the differentiation and expansion of MDSCs and the subsequent inhibition of T-cells. Acceleration of STING degradation by Gal-9 inside tumor cells is one key step of this process.

The present study reveals that in NPC cells, Gal-9 significantly up-regulates the genes encoding cytokines and chemokines related to myeloid cell differentiation and expansion, including IL-1β, IL-1α, IL-6, CXCL8, CX3CL1, CCL22, and CCL-5. While IL-1β and IL-6 have been linked to the generation of MDSC inside tumor tissues, CX3CL1 and CCL22 have been found to induce the recruitment of MDSCs into the tumor microenvironment in tumor-bearing hosts^34–36^. The data from previous reports on the effects of Gal-9 on the differentiation and function of innate immune cells are not entirely consistent. In a small number of cases, Gal-9 has been shown to stimulate innate immunity and play a protective role against microbial infection or tumor progression^37–39^. However, other studies have highlighted mechanisms of immune suppression involving cells of the innate immune system. Golden-Mason et al. have reported functional impairment of human NK cells by extra-cellular Gal-9^40^. Using transgenic mice Dardhalon et al. have shown that systemic overexpression of Gal-9 results in the expansion of splenic CD11b^+^Ly-6G^+^ MDSCs^9^. More recently, Zhang et al. have reported a similar effect obtained by IP or IV injections of recombinant Gal-9 in a murine model of myocarditis^41^. Here, for the first time, we report a stimulating effect of Gal-9 on human MDSCs. We demonstrate that Gal-9 expressed inside malignant cells maximizes the induction of MDSCs in their microenvironment, leading to myeloid cell-mediated T cell inhibition. In addition, our findings support the concept that extracellular soluble or exosomal Gal-9 induces differentiation of CD33^+^CD11b^+^HLA-DR^−^MDSCs from CD33^+^ cells through their production of cytokines IL-1β and IL-6, which have been linked to MDSC differentiation^42^. Moreover, we have shown in our previous studies that IL-1β and IL-6 are up-regulated in NPC cells by LMP1-induced glycolysis or suppression of STING signals, leading to tumor-associated MDSC expansion^10,22^. Therefore, here our results suggest that intracellular and extracellular Gal-9 promotes MDSC differentiation and expansion through enhanced production of suppressive cytokines and chemokines such as IL-1β and IL-6 from both tumor cells and CD33^+^ cells (see our tentative synthesis in Fig. 7).

The STING signaling pathway has emerged as a central TLR-independent mediator of host innate defense stimulated by cytosolic nucleic acids from foreign microbial infection^43^. Emerging evidence suggests that the STING pathway represents a central node in bridging innate immunity and subsequent adaptive immunity in virus infection and cancers^44^. Our and others’ recent studies demonstrated that inactivation of the STING pathway is linked to MDSC expansion in both human and murine tumor model^21,22^. We have previously reported that STING restrains MDSC differentiation by decreasing IL1β and IL-6 production from NPC or CD33^+^ cells through suppressing the SOCS1/STAT3 signaling pathway^22^. Here we have been a step further by showing that Gal-9 could suppress STING expression in NPC cells and myeloid cells, and that deactivation of STING signaling is required for Gal-9-mediated MDSC expansion.

In virus-associated tumors like EBV- and HPV-positive carcinomas, the virus products, including viral DNA molecules, usually trigger antivirus signals especially TLR and STING signaling pathways. Accordingly, the tumor cells have to restrain these antivirus pathways to escape the host immune surveillance^23^. Here, our data show that Gal-9 negatively regulates STING stability through protein-protein binding in both tumor cells and CD33^+^ cells. In addition, we found that the interaction of Gal-9 and STING enhance the TRIM29-mediated K48-linked ubiquitination of STING. It has been reported that human airways epithelial cells selectively express TRIM29 and that its expression can be further induced by EBV infection. In turn, TRIM29 induced K48-linked ubiquitination of STING for protein degradation, which enhances EBV-positive tumor cell survival^23^. Here, our data further suggest that Gal-9 enhances the interaction of STING and TRIM29 to accelerate the degradation of the STING protein. The mobilization of the Gal-9/TRIM29/STING axis results in myeloid-cell-mediated T-cell suppression, which is beneficial for EBV-positive tumor cell survival. Importantly, our observations highlight a novel molecular mechanism explaining STING down-regulation in tumors with abundant Gal-9 production. The role of Gal-9 in auto-immune diseases is probably variable depending on the tissue and pathological context. For example, depending on the publications, Gal-9 has been reported either to increase or, on the contrary, to decrease the severity of SLE (systemic lupus erythematosus) in patients and in murine models^45–47^.

In NPC patients, we observed that Gal-9 was abundant in the tumor cells by comparison with the surrounding non-tumor tissue and in the serum by comparison with serum samples from healthy individuals (Fig. 6a, b). Moreover, the abundance of Gal-9 in tumor cells and serum samples was indicative of a pejorative outcome in terms of DFS and OS. High expression of Gal-9 in tumor cells was correlated with the risk of relapse but not with the tumor stage whereas serum concentration of Gal-9 was correlated with both parameters. To explain these differences, we speculated that Gal-9 serum concentration is probably more dependent on the total number of producing cells than on the average cellular amount of Gal-9. In other words, it might be more dependent on the total tumor volume which can vary by at least one order of magnitude from one patient to another. One major aim of our future studies on NPC specimens will be to seek correlations between Gal-9 abundance in tumor cells and/or in serum samples and the abundance of MDSCs in the tumor leucocyte infiltrate as well as in the PBMCs. This will help us to determine the potential of circulating Gal-9 as a tumor biomarker.

Our findings highlight a novel mechanism involving Gal-9 and supporting cytokine-induced expansion of MDSCs to create a tumor suppressive microenvironment, which favor tumor progression (Fig. 7). They point to Gal-9 as a possible physiological partner of STING in the regulation of innate immunity.

## Materials and methods

### Patient samples and cell lines

Paraffin-embedded tumor tissues (*n* = 92) and serum samples (*n* = 93) were collected from 117 NPC patients at the Sun Yat-Sen University Cancer Center, Guangzhou, China from 2011 to 2015 (Supplementary Tables S1 and S2). Tumor-adjacent tissues were collected from 14 of 92 NPC patients with tumor tissue samples and both tumor specimens and serum samples were obtained from 68 of 117 NPC patients. Twenty healthy donors were included as controls. All patients and healthy donors signed a consent form approved by the Research Ethics Committee of the Sun Yat-sen University Cancer Center (GZR2013-040). This study was performed in accordance with the Helsinki Declaration.

HEK293T, TW03, and C666-1 cells were cultured in DMEM (Life Technologies) or RPMI-1640 medium (Life Technologies) supplemented with 10% fetal bovine serum (Gibco) and 1% L-glutamine (Gibco). All cell lines were tested Mycoplasma-free as determined by PCR-based method (16s rDNA-F: 5′-ACTCCTACGGGAGGCAGCAGTA-3′, 16s rDNA-R: 5′-TGCACCATCTGTCACTCTGTTAACCTC-3′). Mycoplasma testing was carried out every 2 weeks, and the cells were not cultured for more than 2 months.

### RNA extraction, RNA-seq and quantitative RT-PCR (qRT-PCR) analysis

Total RNA was isolated with Trizol reagent (Invitrogen) according to the manufacturer’s protocol. The concentration and quality control of total RNA were determined in triplicate with a NanoDrop2000 spectrophotometer (Thermo Scientific). RNA sequencing was performed with poly(A)-enriched mRNAs at Guangzhou RiboBio Company. Biological pathway analysis was performed using the Kyoto Encyclopedia of Genes and Genomes (KEGG, http://www.genome.jp/kegg/pathway.html), and the selective pathway program for the significantly different gene expression profiles was drawn using KOBAS 2.0 software (http://kobas.cbi.pku.edu.cn/index.php). Raw read counts per loci were normalized across samples, and differential gene expression was analyzed using DESeq. The RNA-seq data were deposited in the NCBI Gene Expression Omnibus under accession code GSE125942. Quantitative RT-PCR was performed with the SYBR Green qPCR Mix (GenStar), the detailed methods have been previously described^22^. Specific primers for qPCR are listed in Supplementary Table S3.

### Fluorescence-activated cell sorting (FACS) analysis

For FACS analysis, single-cell suspensions were stained with fluorescent antibodies according to the manufacturer’s instructions. Fluorescent antibodies conjugated with different fluorescent dyes and matched isotypes were obtained from eBioscience: including CD4 (555346); CD8 (12-0088-42); CD11b (85-25-0118-42); CD33 (85-45-0338-42), HLA-DR (85-12-9952-42) and IFN-γ (17-55-7319-82). In brief, cells were harvested, washed and stained with surface phenotypic markers for 20 min on ice. After permeabilization and fixation, cells were intracellularly stained with IFNγ-APC antibodies. Positively stained cells were detected using a Beckman Coulter Gallios Flow Cytometer and analyzed using the Flowjo V10 software.

### Immunoblot and Immunoprecipitation (IP) assays

For immunoblot assays, NPC or HEK293T cells were subjected to lysis in ice-cold low-salt lysis buffer (LSB, 150 mM NaCl, 50 mM Hepes pH 7.5, 1.5 mM MgCl_2_,1 mM EDTA, 10% glycerol, 1% Triton X-100) supplemented with 5 mg/mL protease inhibitor cocktail (Roche, Basel, Switzerland). Aliquots of 20–25 μL extracts were subjected to SDS-PAGE. For IP assays, NPC or HEK293T cells were transfected with corresponding expression vectors for indicated times, then Flag- or Myc-tagged proteins were pulled down using Flag-beads (Sigma -Aldrich, St. Louis, MO, USA) or Myc-beads (Immuoway, Newark, DE, USA). The following antibodies were used which were directed to: FLAG (Sigma, A8592), β-actin (Sigma, A2228), HA (Roche Applied Science, clone 3F10), Myc (Roche Applied Science, 11814150001), STING (Proteintech, IL, USA, 19851-1-AP), TRIM29 (Proteintech, 17542-1-AP).

### Statistical analysis

All analyses were performed using GraphPad Prism 5 software (La Jolla, CA, USA) and SPSS 18.0 software (Chicago, IL, USA). Numbered results were expressed as mean ± standard error of the mean (SEM). Two-tailed Student’s *t* test was used for comparison of the numerical data. The Kaplan–Meier and log-rank test were used for survival analysis, and a *χ*^2^ test was also used in some experiments as indicated. For IHC scores, cutoff values were the median of each group. In this study, *p < 0.05, **p < 0.01, ***p < 0.001, and *p* < 0.05 was considered significant. The authenticity of this article has been validated by uploading the key raw data onto the Research Data Deposit (RDD) public platform (www.researchdata.org.cn), with the RDD approval number RDDB2019000748.

## Supplementary information

Supplement figure legends

Supplemental Materials and Methods

Tables S1-S3

Figure S1

Figure S2

Figure S3

Figure S4

Figure S5
