# Correction to: Galectin-9 promotes a suppressive microenvironment in human cancer by enhancing STING degradation

**DOI:** 10.1038/s41389-021-00375-2

**Published:** 2022-01-19

**Authors:** Chuan-xia Zhang, Dai-jia Huang, Valentin Baloche, Lin Zhang, Jing-xiao Xu, Bo-wen Li, Xin-rui Zhao, Jia He, Hai-qiang Mai, Qiu-yan Chen, Xiao-shi Zhang, Pierre Busson, Jun Cui, Jiang Li

**Affiliations:** 1 Department of Biotherapy, State Key Laboratory of Oncology in South China, Collaborative Innovation Center for Cancer Medicine, Guangdong Key Laboratory of Nasopharyngeal Carcinoma Diagnosis and Therapy, Sun Yat-sen University Cancer Center, and School of Life Sciences, Sun Yat-sen University, 510060 Guangzhou, P. R. China; 2 MOE Key Laboratory of Gene Function and Regulation, State Key Laboratory of Biocontrol, Sun Yat-sen University, 510275 Guangzhou, China; 3 Department of Nasopharyngeal Carcinoma, Sun Yat-sen University Cancer Center, 510060 Guangzhou, China; 4 CNRS, UMR 9018, Gustave Roussy and Université Paris-Saclay 39 rue Camille Desmoulins, F-94805 Villejuif, France; 5 Department of Neurosurgery, The First People’s Hospital of Changzhou, Changzhou, 213000 Jiangsu China; 6 Department of Research and Development, Shenzhen Institute for Innovation and Translational Medicine, Shenzhen International Biological Valley-Life Science Industrial Park, Dapeng New District, Shenzhen, China

**Keywords:** Cancer microenvironment, Tumour immunology

Correction to: *Oncogenesis* 10.1038/s41389-020-00248-0, published online 06 July 2020

Following the publication of this article the authors noted errors in Figs. 2 and 5.

In Fig. 2e, the FACS plot image was duplicated by mistake.

In Fig. 5B, an additional sample was included in the lower β-actin band by mistake.

Both figures have now been corrected

The authors confirm that these errors do not affect the conclusions of the article.

The original article has been corrected.

