# Supplementary material for: Galectin-9 promotes a suppressive microenvironment in human cancer by enhancing STING degradation
Source: Oncogenesis. 2020 Jul 6;9(7):65. doi: 10.1038/s41389-020-00248-0 (PMC7338349; doi:10.1038/s41389-020-00248-0)
Supplement: Supplementary file 1 — Supplement figure legends [file 41389_2020_248_MOESM1_ESM.docx]

**Supplementary Figure legends**

**Supplementary Fig. S1 Forced Gal-9 expression influences the gene expression profile related to innate immune cell differentiation in NPC cells.** (**a**) Protein levels of Gal-9 in NPC cell lines, including CNE1, CNE2, TW03 and C666-1 cells. (**b** and **c**) qPCR analysis (upper panel) and immunoblot analysis (lower panel) of Gal-9 expression in Gal-9 TW03 cells (**b**) and shGal-9-01 and shGal-9-02 C666-1 cells (**c**). (**d**) A listing of representative KEGG pathways (Kyoto Encyclopedia of Genes and Genomes, <http://www.genome.jp/kegg/pathway.html>) associated with those differentially expressed genes in TW03-Gal-9 *vs* TW03-EV cells. (a-c) were performed at least three times, and the quantification data were plotted as the mean ± SEM.

**Supplementary Fig. S2** **Extracellular Gal-9 directly promotes MDSC differentiation.** (**a**) The lysates of Exo-TW03-EV, Exo-TW03-Gal9, Exo-C666-1-shCtrl and Exo-C666-1-shGal-9 were subjected to immunoblot with anti-Gal-9 and anti-CD63 antibodies. (**b**) Representative (left) and quantification (right) of flow cytometry plot of MDSC differentiation assay are shown as the CD33^+^CD11b^+^ cells in the HLA-DR^-^ gate induced by Exo-TW03-EV, Exo-TW03-Gal9, Exo-C666-1-shCtrl and Exo-C666-1-shGal-9. (**c**) ELISA assay of IL-6 and IL-1β levels in the supernatants from CD33^+^ cells infected with Exo-TW03-EV, Exo-TW03-Gal9, Exo-C666-1-shCtrl and Exo-C666-1-shGal-9. All experiments were performed at least three times, and the data were plotted as the mean ± SEM. Statistics were conducted with an unpaired Student’s t test, *p < 0.05, **p < 0.01 versus the corresponding control. Exo, exosomes. NS, not significant.

**Supplementary Fig. S3 Generating STING knock out TW03 cells.** The lysates of TW03 STING knockout (KO) cells were immunoblotted with an anti-STING antibody.

**Supplementary Fig. S4** **Gal-9 interacts with STING.** The lysates of TW03 cells were transfected with HA-STING, Flag-EV, Flag-Gal-9 and its mutants, were subjected to immunoblot with the indicated antibodies. The experiment was performed at least three times.

**Supplementary Fig. S5** **Gal-9 enhances STING interaction with and ubiquitination by TRIM29.** (**a**) Lysates of TW03 TRIM29 knockout (KO) cells were immunoblotted with an anti-TRIM29 antibody. (**b**) HEK293T cell were transfected with the indicated plasmids, and the lysates were immunoblotted with the indicated antibodies. (**c**) TW03 cells were transfected with Flag-EV, Flag-Gal-9, together with HA-TRIM29 plasmids for 24 h. The cell extracts were subjected to immunoprecipitation with anti-Flag-beads and immunoblot analysis with anti-HA antibody. All experiments were performed at least three times.
