# Supplementary material for: Galectin-9 promotes a suppressive microenvironment in human cancer by enhancing STING degradation
Source: Oncogenesis. 2020 Jul 6;9(7):65. doi: 10.1038/s41389-020-00248-0 (PMC7338349; doi:10.1038/s41389-020-00248-0)
Supplement: Supplementary file 2 — Supplemental Materials and Methods [file 41389_2020_248_MOESM2_ESM.docx]

**Supplemental Materials and Methods**

**Plasmids and Reagents**

All plasmid constructions were verified by sequencing and transfected into cells using Lipofectamine 2000 (Invitrogen) according to the manufacturer’s instructions. Plasmids containing the full coding sequences of various wild type and mutant proteins, including Gal-9, STING, TRIM29, Gal-9-CBD1 (carbohydrate-binding domain-1), Gal-9-CBD2 (carbohydrate-binding domain-2), STING-NT (N-terminal) and STING-CT (C-terminal) were constructed as previously described ([1](#_ENREF_1)). Then they were cloned into Flag-tagged, HA-tagged and Myc-tagged pcDNA 3.1 expression plasmids using classical PCR, which used in transient transfections. Gal-9 gene was also subcloned into a FG-EH-Flag-Dest lentiviral vector (provided by Xiaofeng Qin laboratory). Gal-9 specific shRNA (shGal-9) and control (shCtrl) vectors were constructed using vector pLKO.1. All plasmid constructions were verified by sequencing and transfected into HEK293T cells using Lipofectamine 2000 (Invitrogen). Plasmids containing STING and TRIM29 sgRNAs for CRISPR/Cas9 were constructed as described previously ([2](#_ENREF_2)). The sequence of sgRNA for knock out STING and TRIM29 were as follows:

*STING* sgRNA, 5’- GGTGCCTGATAACCTGAGTA -3’

TRIM29 sgRNA 1#, 5’- GGAACCACATGGAGAACGGT -3’

TRIM29 sgRNA 2#, 5’- GTCTCCTTGGTGAAGCGGCC -3’

TRIM29 sgRNA 3#, 5’- GGAGGAGCTGCCTTGGACGA -3’.

**Lentivirus production and** **Generation of stable expression or knockout cell lines**

For lentivirus production, HEK293T cells were transfected with corresponding vectors (Δ8.9:VSVG:expression vectors =3:2:5) for 48 h, and the supernatants containing lentiviral particles were collected and stored at 4°C until use. For generating stable overexpression or knockout cell lines, NPC cells was infected with serial dilutions of lentivirus in the presence of 8 μg/ml polybrene (Abbott Laboratories). Then, 48 h post-infection, lentivirus-infected NPC cells were cultured with 3 μg/ml puromycin (Sigma-Aldrich) for 2 weeks. The puromycin-resistant colonies were then collected and expanded for further analysis under selective conditions.

**Enzyme-linked immunosorbent (ELISA) assay**

For ELISA assay, human Gal-9 (R&D Systems, Minneapolis, MN, USA), human IL-1β and IL-6 ELISA kits (eBioscience, San Jose, CA, USA) were used in this study，the detailed methods have been previously described (10).For Gal-9 ELISA, according to the manufacturer’s instructions, 96-well plate was coated with 100 μL per well of diluted capture antibody at RT overnight. After washing, the plate was blocked with 200 μL per well reagent diluent at RT for 1 hour. Then 100μl of standard or 4-times-diluted samples were added to each well for 2 hours at RT after another washing. After repeating the washing step, 100 μL per well of the detection antibody was added for another incubation of 2 hours at RT. Then 100 μL of streptavidin-HRP was added into each well for a 20 min incubation in dark after washing. The washing step was repeated again, followed by adding 100 μL per well of substrate solution at RT in dark for 20 min. Then 50 μL of stop solution was added in each well. The detection of absorption at 450 nm was operated with a 96-well plate reader (Bio-Rad).

**Exosome isolation**

Briefly, collected culture supernatants from TW03-EV and TW03-Gal-9 cell supernatants were subjected to differential centrifugation at 900 × g for 15 min at 4°C, 1900 × g for 15 min at 4°C and 12000 × g for 45 min at 4°C to remove large cell debris and large vesicles. The remaining supernatants were then filtered (0.22-μm Millex GP, Darmstadt, Germany) and centrifuged at 120 000 × g, 180 min at 4°C. The exosome pellets were then washed by suspension in 20 ml phosphate-buffered saline (PBS), followed by ultracentrifugation at 120,000 × g for another 180 min at 4°C. Isolated exosomes were resuspended in sterile PBS and maintained at −80 °C for further study.

**Assays of MDSC differentiation and** **IFN-γ-producing T-cell proliferation**

MDSC differentiation assays were conducted as described previously (22). Firstly, using human CD33 MicroBeads (Miltenyi Biotec, Bergisch Gladbach, Germany), CD33^+^ cells were isolated from PBMCs of healthy donors. Secondly, NPC cells were co-cultured with isolated CD33^+^ cells in 24-well plates using a Transwell System (0.4 μm pore, Corning, New York, NY, USA) at a ratio of 1:5 for 2 days. CD33^+^ cells cultured in medium alone were included as controls. The percentage of CD33^+^CD11b^+^HLA-DR^-^ MDSCs were measured by FACS analysis. For the IFN-γ-producing T-cell proliferation assay, in brief, PBMCs were labeled with carboxyfluorescein diacetate succinimidyl ester (CFSE, 10 μM), then plated in OKT3-coated 96-well plates and added to the induced MDSCs at ratio 1:1, followed by culturing for three days. Then the cells were harvested and stained for IFN-γ, CD3, CD4, and CD8 and subjected to analysis by a [Beckman Coulter Gallios Flow Cytometer.](http://www.beckmancoulter.com/wsrportal/bibliography?docname=BR-18063.pdf)

**Immunohistochemistry (IHC) and Immunofluorescence (IF) assays**

For IHC assays, paraffin-embedded tissues were continuously sectioned at a thickness of 4 µm. Immunohistochemical (IHC) staining of Gal-9 was performed using a primary mouse anti-human Gal-9 as previously described ([3](#_ENREF_3)). For IHC, paraffin-embedded tissues were sectioned continuously at a thickness of 4 µm, followed by staining with an indicated antibody according to the manufacturer’s instructions. The data were scored for Gal-9 in tumor nests and adjacent tissues in 5 separate 400× high-power microscopic fields (HPFs). Specimens were scored according to the multiplication of intensity of immunohistochemical staining (0, no staining; 1, weak; 2, moderate; and 3, strong). The slides were scored independently by two pathologists. Cutoff selection was based on median. For IF assays, harvested C666-1 or CD33^+^ cells were incubated with Gal-9, STING or TRIM29 antibodies for 30 min and followed by CF488-conjugated anti-mouse antibody or CF568-conjugated anti-rabbit secondary antibody, and then stained by Hoechst (2 µg/ml) and fixed with 4% paraformaldehyde. Images were acquired using a fluorescence microscope (Leica) and images were observed using ImageJ software (National Institutes of Health).

**References**

1. Zhang CX, et al. STING signaling remodels the tumor microenvironment by antagonizing myeloid-derived suppressor cell expansion. Cell death and differentiation 2019; **26**: 2314-28.

2. Zhang J, et al. TRIM45 functions as a tumor suppressor in the brain via its E3 ligase activity by stabilizing p53 through K63-linked ubiquitination. Cell death & disease 2017; **8**: e2831.

3. Barjon C, et al. A novel monoclonal antibody for detection of galectin-9 in tissue sections: application to human tissues infected by oncogenic viruses. Infectious agents and cancer 2012; **7**: 16.
