# Supplementary material for: Galectin-9 promotes a suppressive microenvironment in human cancer by enhancing STING degradation
Source: Oncogenesis. 2020 Jul 6;9(7):65. doi: 10.1038/s41389-020-00248-0 (PMC7338349; doi:10.1038/s41389-020-00248-0)
Supplement: Supplementary file 3 — Tables S1-S3 [file 41389_2020_248_MOESM3_ESM.docx]

**Table S1**. **The association between the levels of tumor and serum Gal-9 and clinical parameters of patients**

| **Clinical characteristics** | **High level of Gal-9 in tumor tissues (score >2.4)** | | | **High level of Gal-9 in serum (> 2.8ng/mL)** | | |
| --- | --- | --- | --- | --- | --- | --- |
|  | **Total case** | **No. (%)** | **P value** | **Total case** | **No. (%)** | **P value** |
| **Total case** | 92 | 44 (47.8%) |  | 93 | 47 (50.6%) |  |
| **Age** |  |  |  |  |  |  |
| ≤ 45 y | 50 | 21 (42.0%) | 0.222 | 49 | 21 (42.9%) | 0.118 |
| > 45 y | 42 | 23 (54.8%) |  | 44 | 26 (59.1%) |  |
| **Gender** |  |  |  |  |  |  |
| Male | 76 | 39 (51.3%) | 0.144 | 75 | 41 (54.7%) | 0.104 |
| Female | 16 | 5 (31.2%) |  | 18 | 6 (33.3%) |  |
| **Tumor (T)** |  |  |  |  |  |  |
| T1-T2 | 24 | 8 (33.3%) | 0.098 | 25 | 9 (36.0%) | 0.089 |
| T3-T4 | 68 | 36 (52.9%) |  | 68 | 38 (55.9%) |  |
| **Lymph node (N)** |  |  |  |  |  |  |
| N0-N1 | 58 | 28 (48.3%) | 0.910 | 55 | 23 (41.8%) | **0.043*** |
| N2-N3 | 34 | 16 (47.1%) |  | 38 | 24 (63.2%) |  |
| **Clinical stage (tumor extension)** |  |  |  |  |  |  |
| I-II | 19 | 6 (31.6%) | 0.111 | 19 | 4 (21.1%) | **0.004**** |
| III-IV | 73 | 38 (52.1%) |  | 74 | 43 (58.1%) |  |

Note: * means p < 0.05; IHC, Immunohistochemical staining; No., number; cutoff, the median value of each group

**Table S2. Baseline clinical parameters of patients**

| **Characteristics** | **No. (%) of patients** |
| --- | --- |
| **Total cases** | 117 (100%) |
| **Age** |  |
| Mean | 46.14 |
| Range | 22-75 |
| **Gender** |  |
| Male | 97 (82.9%) |
| Female | 20 (17.1%) |
| **Tumor (T)** |  |
| T1 | 2 (1.7%) |
| T2 | 30 (25.6%) |
| T3 | 66 (56.4%) |
| T4 | 19 (16.2%) |
| **Lymph node (N)** |  |
| N0 | 16 (13.7%) |
| N1 | 56 (47.9%) |
| N2 | 36 (30.8%) |
| N3 | 9 (7.7%) |
| **Distant metastasis (M)** |  |
| M0 | 117(100%) |
| M1 | 0 (0%) |
| **Clinical stage (tumor extension)** |  |
| I | 2 (1.7%) |
| II | 24 (20.5%) |
| III | 64 (54.7%) |
| IV | 27 (23.1%) |
| **Relapse** |  |
| Yes | 21 (17.9%) |
| No | 96 (82.1%) |
| **Death** |  |
| Yes | 15 (12.8%) |
| No | 102(87.2%) |

**Table S3: Primers for qPCR**

| RPL13A -Forward | CCTGGAGGAGAAGAGGAAAGAGA |
| --- | --- |
| RPL13A -Reverse | TTGAGGACCTCTGTGTATTTGTCAA |
| Galectin-9- Forward | GGACGGACTTCAGATCACTGT |
| Galectin-9- Reverse | CCATCTTCAAACCGAGGGTTG |
| IL-1α- Forward | AGATGCCTGAGATACCCAAAACC |
| IL-1α- Reverse | CCAAGCACACCCAGTAGTCT |
| IL-1β- Forward | ATGATGGCTTATTACAGTGGCAA |
| IL-1β- Reverse | GTCGGAGATTCGTAGCTGGA |
| IL-6- Forward | AACCTGAACCTTCCAAAGATG |
| IL-6- Reverse | TCTGGCTTGTTCCTCACTACT |
| CX3CL1- Forward | GCCACAGGCGAAAGCAGTA |
| CX3CL1- Reverse | GGAGGCACTCGGAAAAGCTC |
| CXCL8- Forward | ACTGAGAGTGATTGAGAGTGGAC |
| CXCL8- Reverse | AACCCTCTGCACCCAGTTTTC |
| CCL5- Forward | CCAGCAGTCGTCTTTGTCAC |
| CCL5- Reverse | CTCTGGGTTGGCACACACTT |
| CCL22- Forward | ATCGCCTACAGACTGCACTC |
| CCL22- Reverse | GACGGTAACGGACGTAATCAC |
| ISG15- Forward | TCCTGGTGAGGAATAACAAGGG |
| ISG15- Reverse | GTCAGCCAGAACAGGTCGTC |
| ISG54- Forward | GGAGGGAGAAAACTCCTTGGA |
| ISG54- Reverse | GGCCAGTAGGTTGCACATTGT, |
| ISG56- Forward | TCAGGTCAAGGATAGTCTGGAG |
| ISG56- Reverse | AGGTTGTGTATTCCCACACTGTA |
