# Supplementary figures and images for: Galectin-9 promotes a suppressive microenvironment in human cancer by enhancing STING degradation
Source: Oncogenesis. 2020 Jul 6;9(7):65. doi: 10.1038/s41389-020-00248-0 (PMC7338349; doi:10.1038/s41389-020-00248-0)

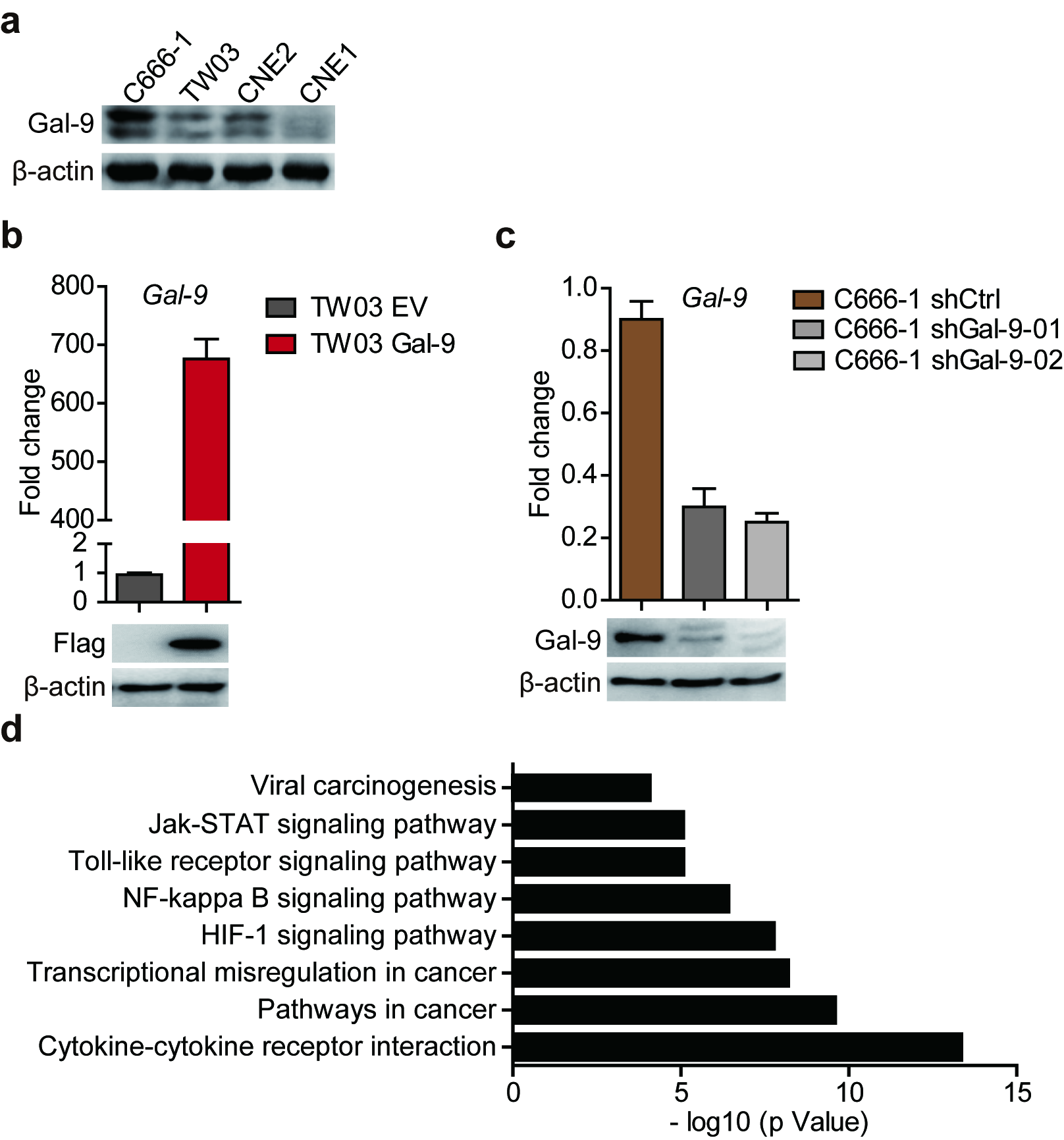

Supplement: Supplementary file 4 — Figure S1 [file 41389_2020_248_MOESM4_ESM.tif]

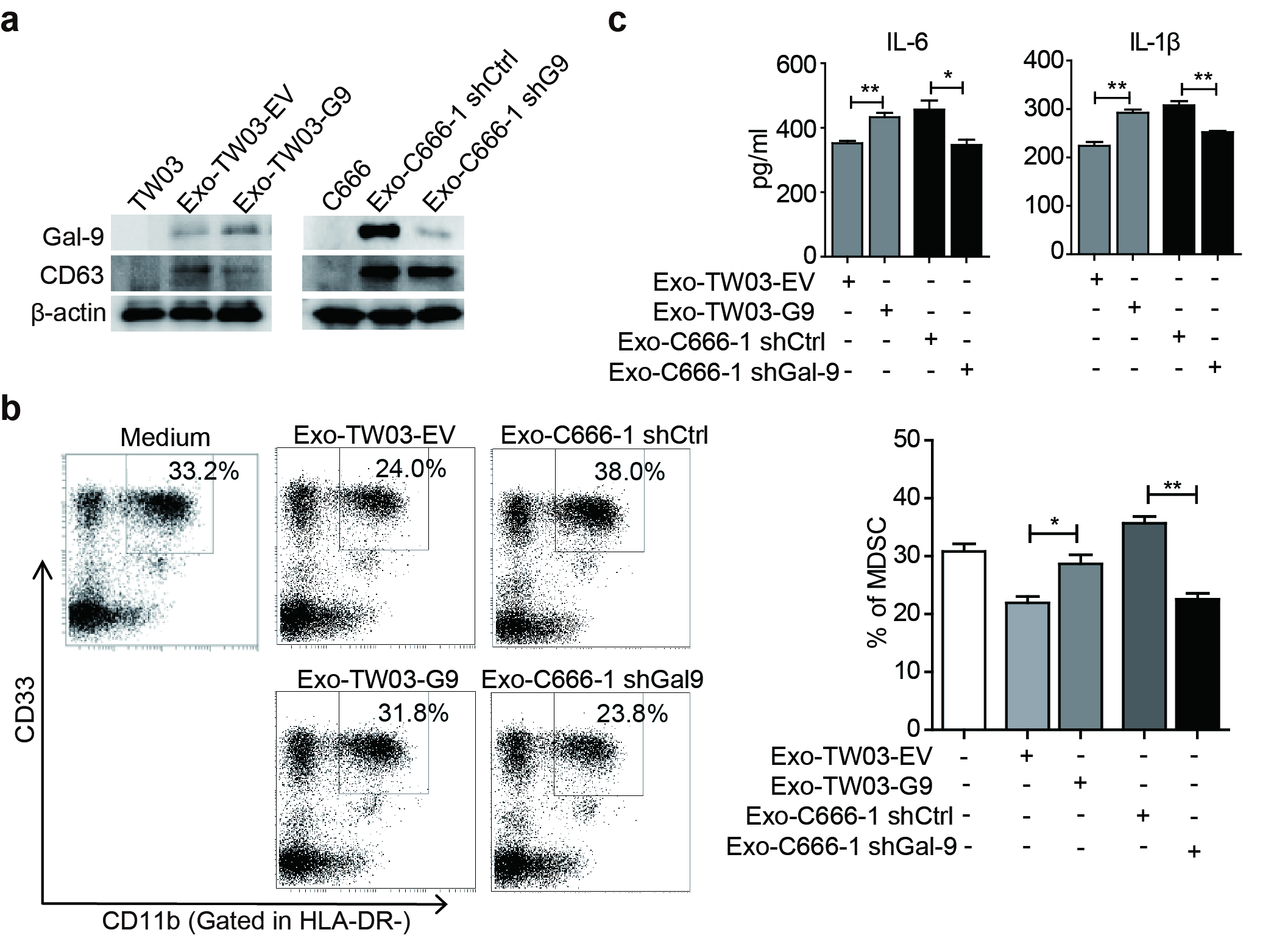

Supplement: Supplementary file 5 — Figure S2 [file 41389_2020_248_MOESM5_ESM.tif]

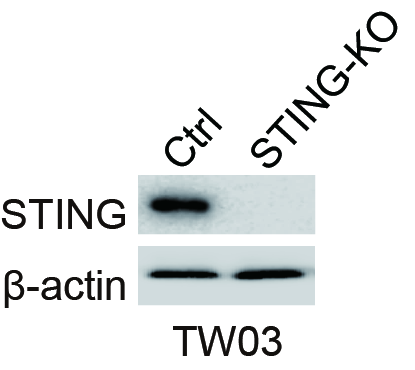

Supplement: Supplementary file 6 — Figure S3 [file 41389_2020_248_MOESM6_ESM.tif]

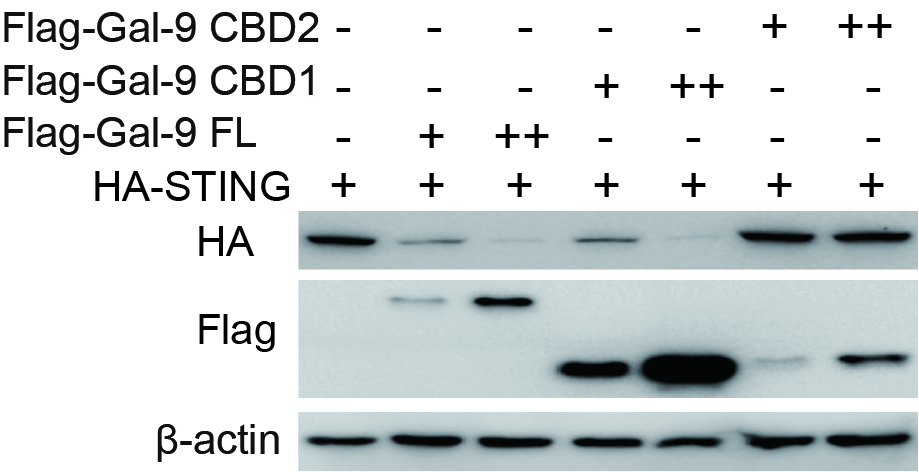

Supplement: Supplementary file 7 — Figure S4 [file 41389_2020_248_MOESM7_ESM.tif]

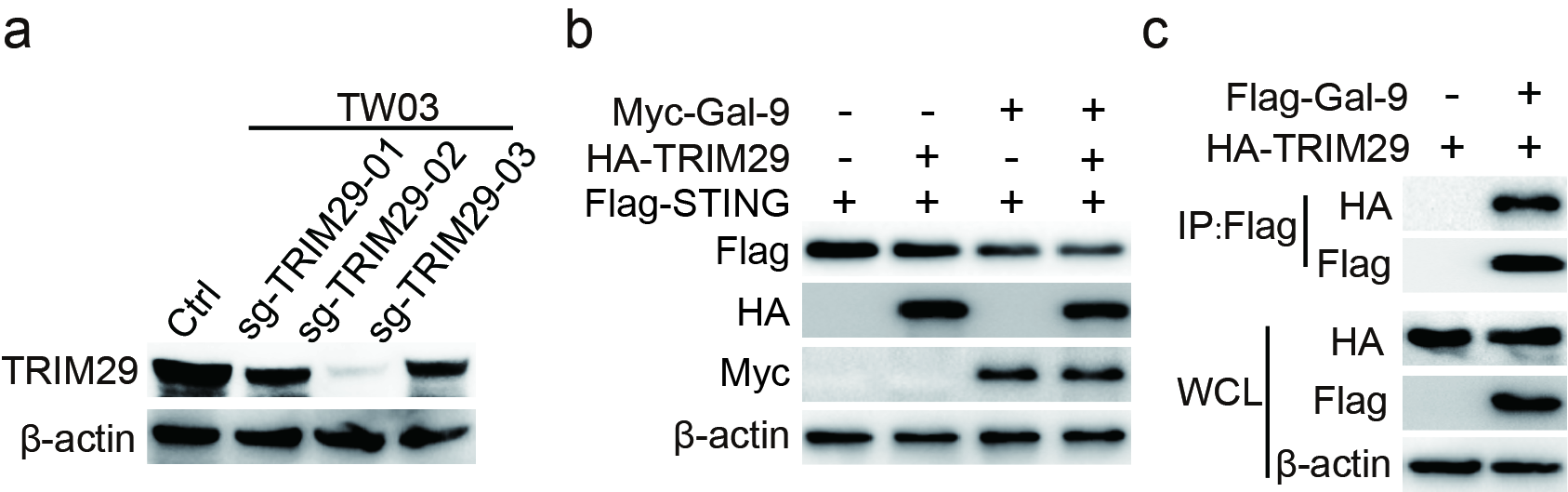

Supplement: Supplementary file 8 — Figure S5 [file 41389_2020_248_MOESM8_ESM.tif]
